# Sox2 acts as a transcriptional repressor in neural stem cells

**DOI:** 10.1186/1471-2202-15-95

**Published:** 2014-08-08

**Authors:** Yu-Ru Liu, Zulfiqar A Laghari, Carolina A Novoa, Jaime Hughes, Jamie RM Webster, Paul E Goodwin, Sally P Wheatley, Paul J Scotting

**Affiliations:** Centre for Genetics and Genomics, School of Life Sciences, Queen’s Medical Centre, Nottingham, NG7 2UH England; Department of Physiology, University of Sindh, Jamshoro, Pakistan; BCCRC, 675 West 10th Avenue, Vancouver, BC Canada

**Keywords:** Sox2, Groucho, Grg, Repressor, Neural stem cells

## Abstract

**Background:**

The transcription factor, Sox2, is central to the behaviour of neural stem cells. It is also one of the key embryonic stem cell factors that, when overexpressed can convert somatic cells into induced pluripotent cells. Although generally studied as a transcriptional activator, recent evidence suggests that it might also repress gene expression.

**Results:**

We show that in neural stem cells Sox2 represses as many genes as it activates. We found that Sox2 interacts directly with members of the groucho family of corepressors and that repression of several target genes required this interaction. Strikingly, where many of the genes activated by Sox2 encode transcriptional regulators, no such genes were repressed. Finally, we found that a mutant form of Sox2 that was unable to bind groucho was no longer able to inhibit differentiation of neural stem cells to the same extent as the wild type protein.

**Conclusions:**

These data reveal a major new mechanism of action for this key transcription factor. In the context of our understanding of endogenous stem cells, this highlights the need to determine how such a central regulator can distinguish which genes to activate and which to repress.

**Electronic supplementary material:**

The online version of this article (doi:10.1186/1471-2202-15-95) contains supplementary material, which is available to authorized users.

## Background

Sox2 is a central player in animal development and one of only a few factors that together can initiate the formation of pluripotent cells (iPS cells) from somatic cell populations [1, 2]. This reflects its central role as a ‘node’ in the gene regulatory network that controls embryonic stem cell biology, promoting stem cell self-renewal and inhibiting differentiation. Sox2 is also one of the first genes to be active in the neural ectoderm and its expression is maintained in proliferating neural stem cells (NSCs) of the CNS throughout development and in the mature brain [3, 4]. The expression of Sox2 in these cells again seems to be associated with their self-renewal and inhibition of differentiation [5]. Sox2 is generally regarded as a transcriptional activator. However, in recent studies analysing the global response of cells to loss of Sox2 activity, the expression of many genes was seen to increase rapidly when Sox2 function was inhibited [6, 7]. ChIP-seq analysis shows that several of the genes affected in these studies are directly bound by Sox2, implying that they are direct targets [8–11]. Given the central role of Sox2 in the biology of both ES cells and NSCs, the possibility that it might also possess such a major alternative mechanism of action is of great interest. We therefore set out to determine to what extent Sox2 represses genes in NSCs. Using an expression array approach, we found that Sox2 repressed approximately as many genes as it activated in NSCs.

We also investigated the mechanism by which Sox2 might achieve transcriptional repression. Tcf and Lef are closely related to the Sox gene family. These factors can act as either transcriptional activators or repressors. Like many other transcriptional repressors, these proteins achieve this effect by recruiting members of the groucho-related gene (Grg) family of co-repressors [12]. We considered this to be a likely route for the repressor activity of Sox2.

We found that Sox2 can indeed interact with Grg proteins and a mutation disrupting interaction between Sox2 and the Grgs resulted in loss of the ability of Sox2 to repress expression from the GFAP promoter whilst its ability to activate other promoters remained intact. We also found that, unlike wild type (WT) Sox2, the mutant version of Sox2 was unable to repress neuronal differentiation when overexpressed. This suggests a new model for the mechanisms by which Sox2 regulates NSC biology.

## Results

### Elucidating the target genes of Sox2 repression

**Figure 1:**
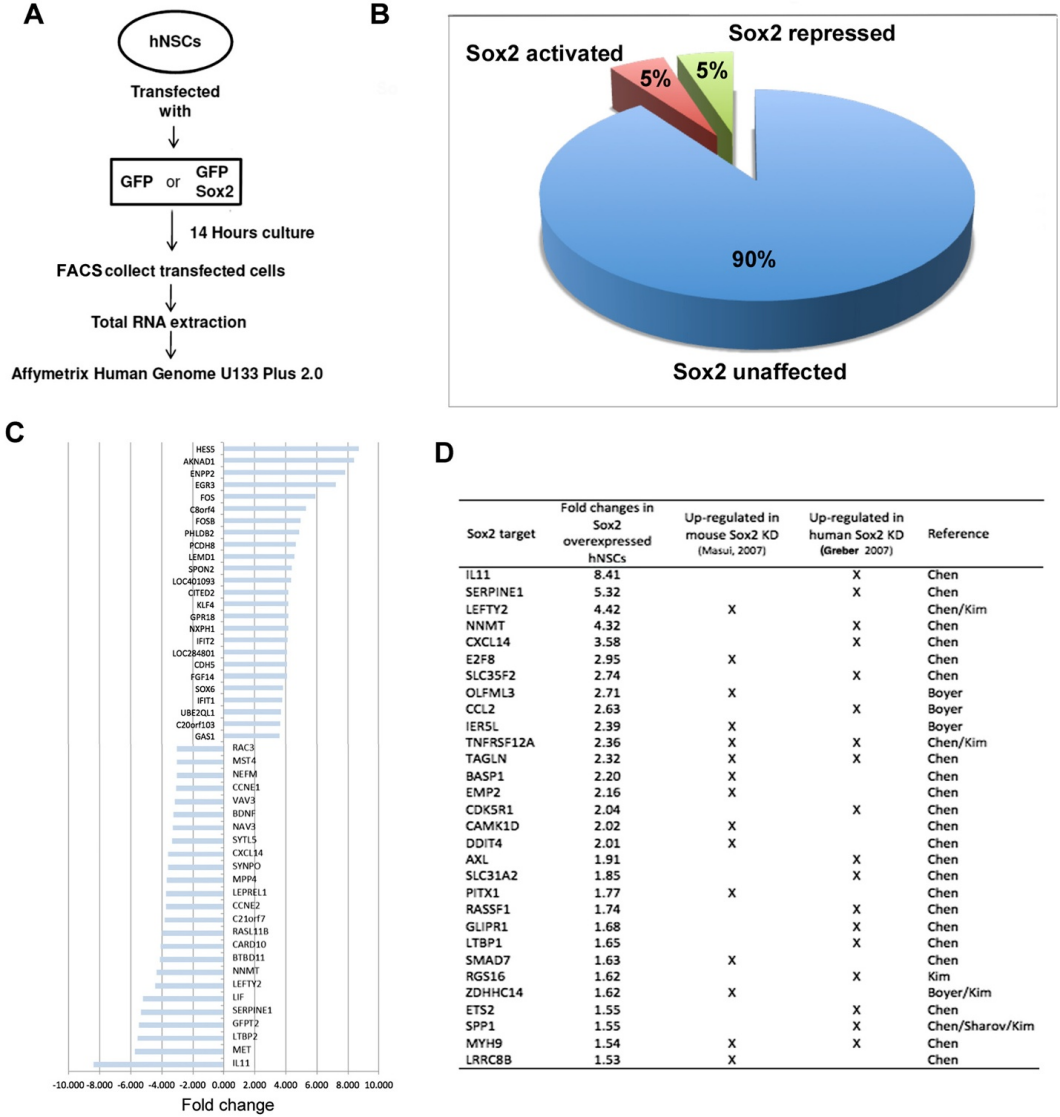
**Potential target genes of Sox2-Grg interaction. (A)** Scheme of the experiment using microarray analysis to identify genes affected by Sox2 in human NSCs. **(B)** The relative percentage of genes affected by Sox2 as compared to control cells transfected by GFP alone (only differences of above 1.5-fold were included in both comparisons). **(C)** Graph representing the most activated (bars to right of midline) and repressed (bars to left of midline) genes. **(D)** The genes most strongly repressed by Sox2 in our study that were also suggested to be repressed by Sox2 in mouse ES cells (Masui et al. [6]), or in human ES cells (Greber et al. [7]). Final column indicates the study in which these were also shown to be direct targets of Sox2.

In order to ascertain the extent to which Sox2 activates and represses genes in NSCs, we carried out gene expression microarray analysis. Human NSCs were transfected with expression constructs encoding EGFP alone, or together with WT Sox2. After 14 hours in culture, EGFP-expressing cells were isolated by FACS and RNA extracted for analysis using Affymetrix Human Genome U133 Plus 2.0 chips (Figure 1A, see Additional file 1: Table S1).

RT-qPCR revealed that the level of Sox2 overexpression was approximately 6 fold greater than endogenous Sox2 and expression of the endogenous Sox2 gene was unaffected in transfected cells. (data not shown). Among the genes that were affected by Sox2 (>1.5 fold change as compared to cells transfected with EGFP alone), the number exhibiting repressed probe sets (650, 5% of genes on the array) was almost the same as the number activated (652) (Figure 1B, C). These numbers are strikingly similar to the number of genes whose expression increased (623) or decreased (648) >1.5 fold when Sox2 expression was lost (using inducible *Sox2*-null mice) in ES cells (Masui et al. [6]).

Comparison of the genes activated by Sox2 to those repressed revealed a striking difference. According to their gene ontology (GO) terms (Using the Gorilla tool [13]) genes that were activated by Sox2 were highly enriched for those listed under terms related to regulating transcription (see Additional file 2: Table S2 and Additional file 3: Table S3). Most of these represent transcription factors. Remarkably, the list of genes repressed by Sox2, included no terms in these same GO term categories. By contrast, among the genes repressed by Sox2, there was very strong enrichment for genes associated with the cell cycle and mitosis, including spindle organization and DNA replication and repair. Such a dramatic difference in the classes of gene activated or repressed by Sox2 provides additional assurance that the genes identified are a non-random selection of the genes on the array.

Previous studies in which Sox2 was knocked down were carried out in ES cells and so would not be expected to share much in common with our experiments in NSCs. However, comparison between these data revealed a small proportion of the genes identified as repressed by Sox2 in our study that were also repressed by Sox2 in ES cells. Some of these genes have also been shown to be bound by Sox2 by ChIPseq (Masui et al. [6]; Greber et al. [7]) (Figure 1D).

### Sox2 interacts with Grg proteins

**Figure 2:**
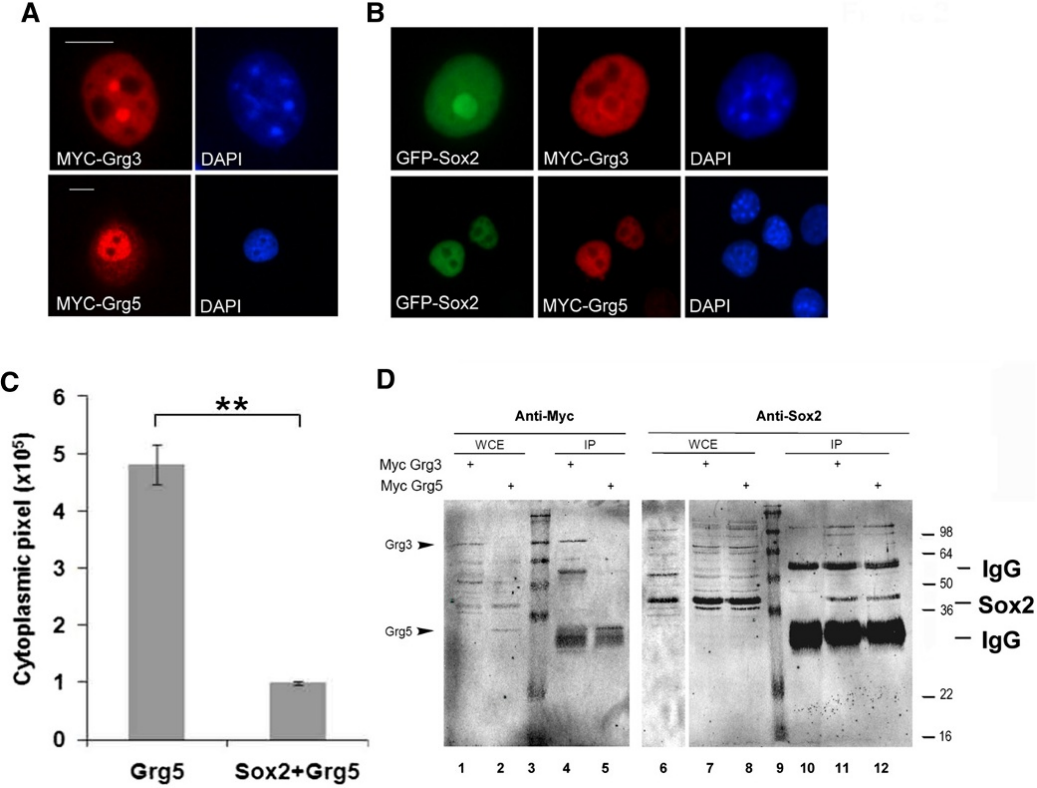
**Sox2 interacts with Grg proteins. (A)** subcellular localization of overexpressed MYC-tagged Grg3 or Grg5 in COS-7 cells; immunostaining with anti-Sox2 or anti-MYC antibody. Nuclei were stained with DAPI. **(B)** Co-transfection of Sox2 with either Grg3 or Grg5, caused the subcellular distribution of the Grgs (red) to alter to reflect that of Sox2 (green). **(C)** Quantification of cytoplasmic Grg5 using Image J software shows effect of Sox2 on Grg5 subcellular distribution was highly significant (p = 0.0023). **(D)** Immunoprecipitation of overexpressed MYC-tagged Grg3 or Grg5 in human neural stem cells, after treatment with DPS crosslinker, resulted in co-precipitation of endogenous Sox2. Left hand blot was probed for the Grgs using anti-MYC, right hand panel probed for Sox2 using annti-Sox2 antibody. WCE - whole cell extract. Size markers in lanes 3 and 9, sizes shown to right of image. Scale bar in for panel A and B approximately 10 μm.

Other HMG domain factors have been shown to interact with the Grg family of co-repressors [14, 15]. Therefore, in order to determine if this might also be a potential mechanism by which Sox2 could act as a transcriptional repressor, we used two assays. First, we determined if Sox2 could alter the subcellular localization of Grg proteins. There are five Grg genes in vertebrates; Grg1-4 are long forms and Grg 5 is equivalent to only the N-terminal half of those long forms. We analysed Grg5 and Grg3 as a representative full length Grg protein. When transfected into COS-7 cells, Grg3 forms nuclear bodies, Grg5 is seen in both the nucleus and the cytoplasm and Sox2 exhibits diffuse staining restricted to the nucleus (Figure 2A, see Additional file 4: Figure S1A). When co-transfected with Sox2, both Grg3 and Grg5 adopted exclusively diffuse nuclear staining matching the distribution of Sox2, suggesting an interaction between Sox2 and Grgs (Figure 2B, C). In a second assay, co-immunoprecipitation was carried out using human NSCs, (which are known to express Sox2) transfected with MYC-tagged Grgs. This showed that endogenous Sox2 was co-precipitated with the MYC-tagged Grg3 and Grg5 implying that they exist in a protein complex (Figure 2D).

### Grgs affect Sox2 function

**Figure 3:**
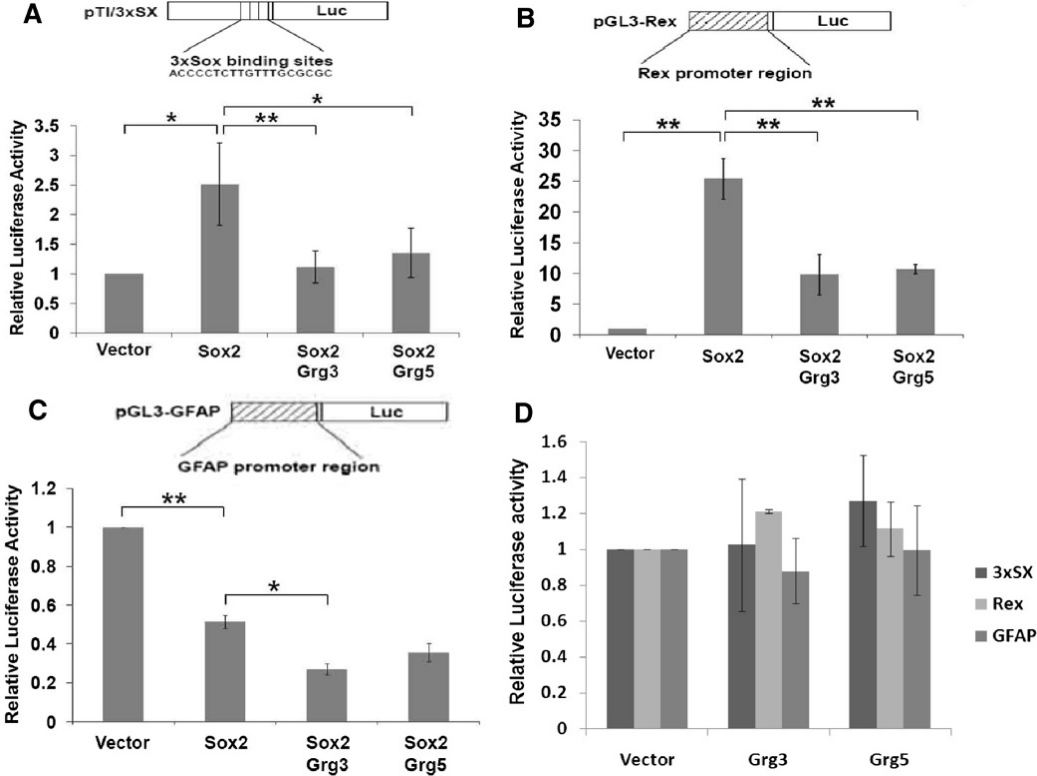
**Grgs repress Sox2 transcriptional activation activity.** Co-transfected Sox2 caused an approximately 2-fold increase in luciferase activity from the 3xSX promoter **(A)** or 25-fold increase from the *REX* promoter **(B)** in Cos-7 cells, but this increase was significantly inhibited when Grg3 or Grg5 were co-transfected **(A,B)**. **(C)** Co-expression of Sox2 with the GFAP-luciferase reporter construct in P19 cells caused a 2-fold decrease in luciferase activity compared to vector alone and the presence of either Grg3 or Grg5 increased this repression (although the difference was only significant in the presence of Grg3). **(D)** Cotransfected Grg3 or Grg5 caused no significant difference from controls (vector) in luciferase activity from various promoters in P19 cells. *p = <0.05; **p = <0.01.

To determine if Grgs affect the transcriptional regulation activity of Sox2, luciferase reporter assays were carried out using several promoter sequences. Co-transfection of COS-7 cells with Sox2 resulted in a twofold increase in luciferase expression from a ‘generic’ Sox promoter (pTl/3xSX, which has three Sox binding sites upstream of the luciferase reporter gene) and a 25-fold increase when the luciferase gene was under the control of the *REX* gene proximal promoter element, a known target of Sox2 [16]. When Grg3 or Grg5 constructs were also transfected alongside Sox2, these increases in luciferase activity were almost completely abrogated or severely reduced (Figure 3A, B). According to Cavellaro et al. [17], expression of the GFAP gene is directly repressed by Sox2. Here, we confirmed this in P19 EC cells, in which the basal level of luciferase activity was significantly repressed by co-transfected Sox2 (Figure 3C). This repression was even greater in the presence of co-transfected Grg3 or Grg5 (Figure 3C), where transfection of the same amount of Grg3 or Grg5 alone had no significant effect on the luciferase expression (Figure 3D).

### Mapping the Grg interaction domain in Sox2

**Figure 4:**
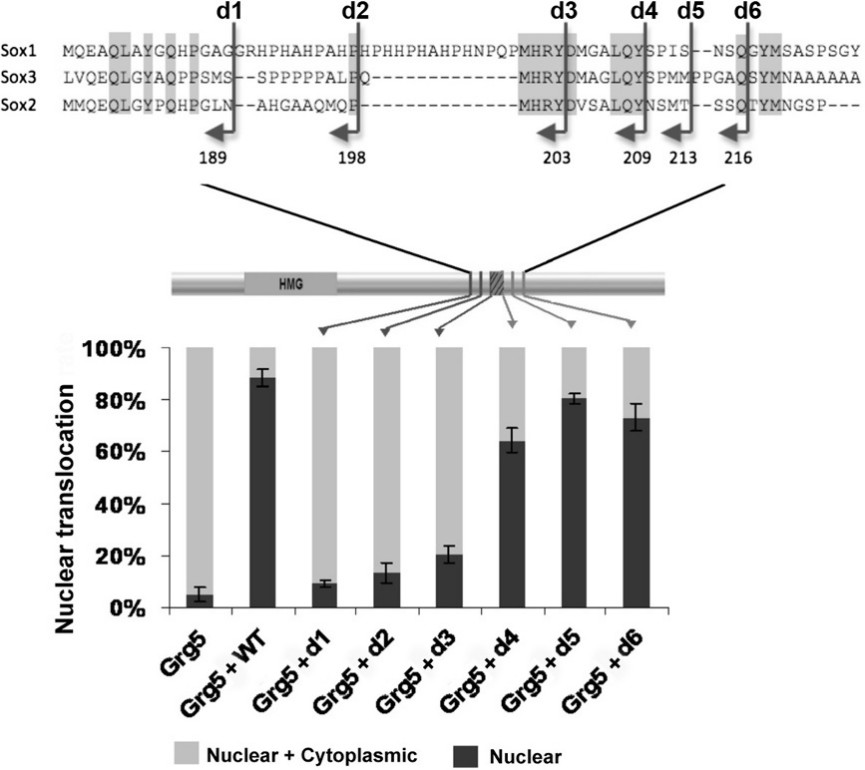
**Mapping the Grg interacting region of Sox2.** Schematic representation of C-terminal deletions and their effect on the ability of Sox2 to cause MYC-tagged Grg5 to translocate to the nucleus. The reduction for d1, d2, and d3 was much greater (p < 0.001) than for d4, d5 and d6 (P = 0.01-0.05).

We designed six C-terminal deletions of Sox2 (Figure 4A) and tested their ability to interact with Grgs (all the constructs produced Sox2 protein that still located to the nucleus, see Additional file 4: Figure S1). The subcellular translocation assay with Grg5 indicated that the C-terminal amino acids between 203 and 209 were essential for the interaction (Figure 4A). We therefore set out to generate a site-directed mutant that no longer bound Grgs, but would retain transcriptional activator activity.

### Loss of Grg interaction in a 203–209 mutant

**Figure 5:**
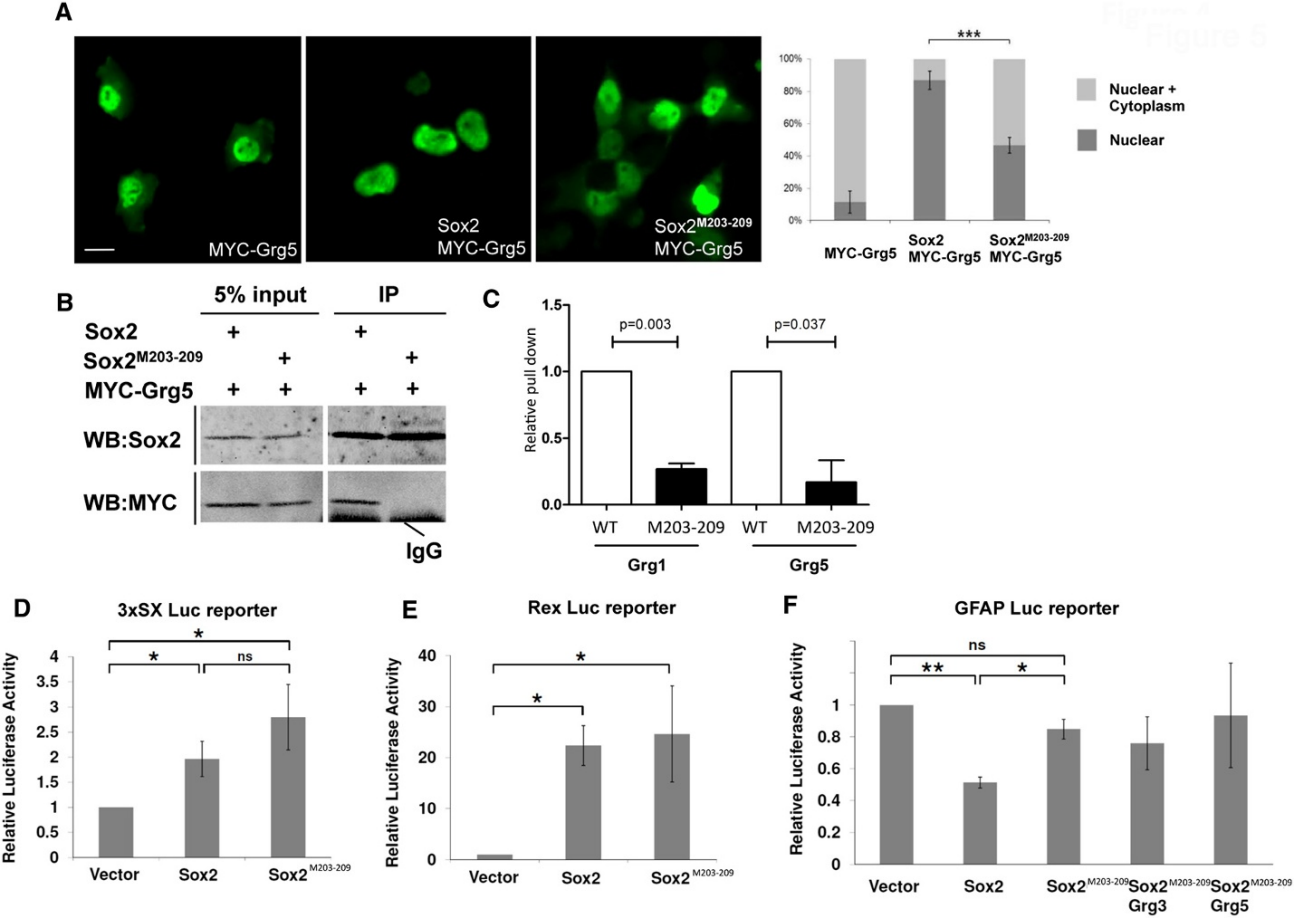
**The Sox2** ^**M203–209**^ **mutant does not interact with Grgs and fails to repress a reporter construct. (A)** Unlike WT Sox2, the Sox2 ^M203–209^ mutant was unable to translocate Grg5 into the nucleus of co-transfected Cos-7 cells suggesting loss of interaction. Counting the proportion of cells exhibiting altered distribution of MYC-Grg in cells co-expressing Sox2 revealed a highly significant reduction of about 50% (p = 0.0002). **(B)** Immune precipitation of MYC-Grg5 co-transfected with WT Sox2 or with the Sox2 ^M203–209^ mutant, showing that the mutant failed to be co-precipitated with Grg5. **(C)** In an *in vitro* pull down assay, immunoprecipitation of GST-fused Grg1 or Grg5 reproducibly pulled-down 4–5 fold less Sox2 ^M203–209^ than it did WT Sox2 **(D)** The Sox2 ^M203–209^ mutant retained the ability to activate luciferase expression driven by the 3xSX promoter in Cos-7 cells (the difference between this and the level induced by WT Sox2 was not statistically significant). **(E)** The ability of co-transfected Sox2 ^M203–209^ mutant to induce luciferase expression driven by the REX promoter in P19 cells was very similar to that induced by WT Sox2. **(F)** Unlike WT Sox2, the Sox2 ^M203–209^ mutant failed to repress luciferase expression driven from the GFAP promoter, and the addition of Grg3 or Grg5 had no effect on this. *p = <0.05; **p = <0.01; ns – not significant Scale bar approximately 20 μm.

Comparison between Sox2 and the other SoxB1 members and between Sox2 orthologues from different species revealed that only four amino acids were conserved in the region from position 203–209. We therefore made a mutant, referred to as Sox2^M203–209^, in which these four amino acids were altered (DxxxLQY converted to VxxxAAA; Figure 4A). Although this mutant still located to the nucleus (see Additional file 4: Figure S1A ), in the subcellular translocation assay, the Sox2^M203–209^ mutant exhibited a dramatically reduced ability to change the subcellular localization of Grg5 (Figure 5A). Moreover, unlike WT Sox2, immune precipitation of MYC-Grg5 in COS-7 cells did not co-precipitate co-transfected Sox2^M203–209^ mutant (Figure 5B). In order to determine whether this mutation had disrupted a direct protein-protein interaction between the Grg proteins and Sox2 an *in vitro* pull down assay was used. The ability of GST-fused Grg1 or Grg5 proteins to pull down radiolabelled Sox2 or Sox2^M203–209^ was assessed. For both Grg proteins, the Sox2^M203–209^ mutant resulted in a similar 4-fold decrease in the amount of Sox2 that was co-immune precipitated (Figure 5C). It was also noted that Grg1 was able to pull down approximately 4-fold more Sox2 than did Grg5 (data not shown). These data show that residues 203–209 of the Sox2 protein are necessary for a direct interaction with both Grg1 and Grg5.

### The Sox2^M203–209^ mutant loses repressor activity

Having established that amino acids 203–209 were required for Grg binding, we next asked whether Sox2^M203–209^ was defective in its ability to act as a gene repressor. Using the luciferase reporter assay described above, we found that the Sox2^M203–209^ mutant retained the ability to activate both the 3xSX and the *REX*-regulated reporter constructs (Figure 5D, E), indeed having a slightly stronger activator effect on the 3xSX promoter than WT Sox2. However, the mutant no longer significantly repressed luciferase expression of the construct driven from the GFAP promoter even in the presence of ectopic Grg3 or Grg5 (Figure 5 F). These results suggest that despite its inability to bind Grg co-repressors, Sox2^M203–209^ retains the transcriptional activator activity of WT Sox2, but is significantly impaired in its ability to repress transcription from the GFAP regulatory sequence.

**Figure 6:**
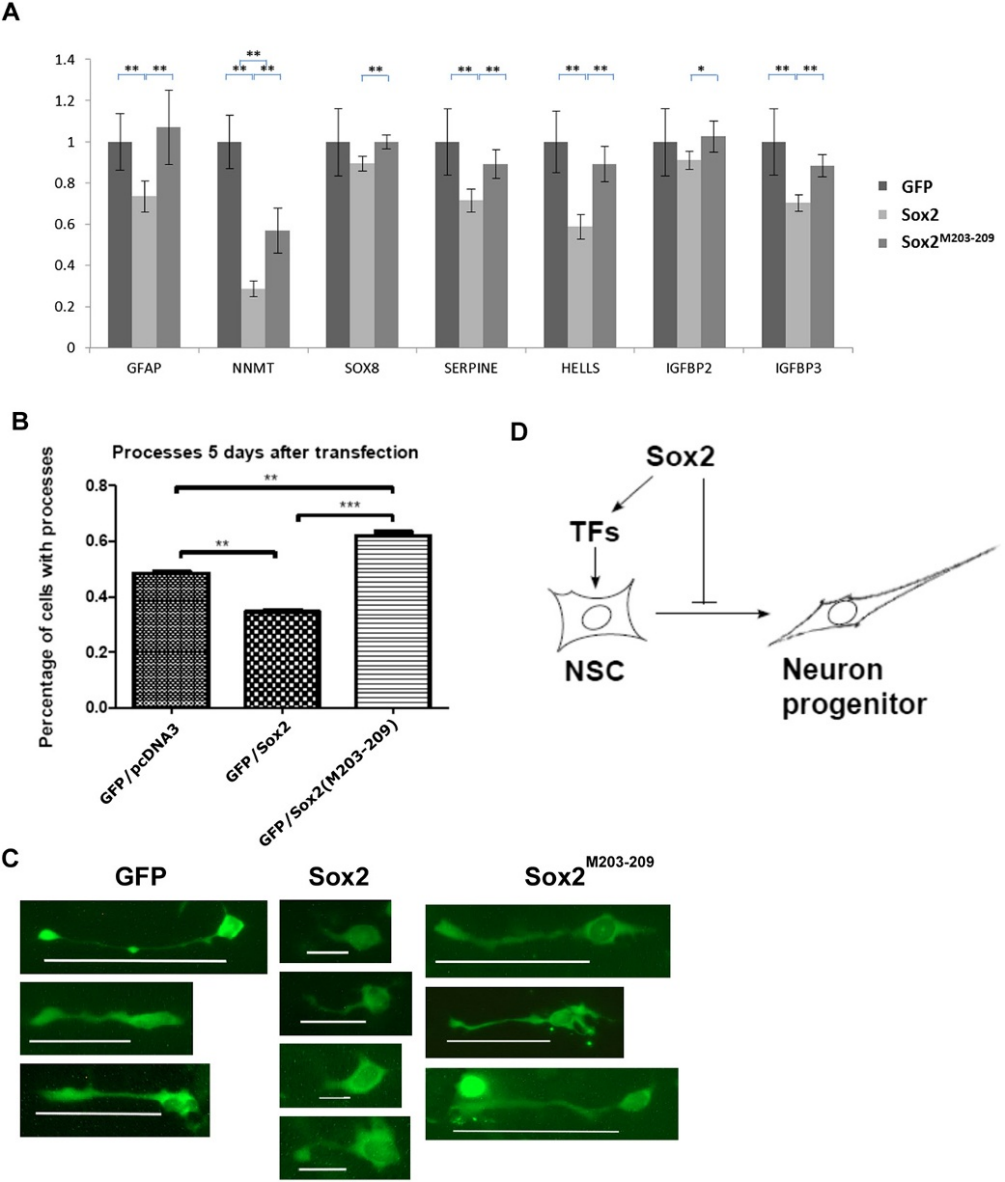
**A Grg binding mutant of Sox2 fails to inhibit NSC differentiation. (A)** Validation of the repression of *NNMT, SOX8, SERPINE1, HELLS, IGFBP2, IGFBP3* and *GFAP* expression using qPCR. P-values shown for 5 PCR replicates *p = <0.05; **p = <0.01. **(B)** Graph showing the percentage of NSCs extending MAP2 positive processes after 5 days in differentiation medium. This was inhibited by WY Sox2 but not by the Sox2 ^M203–209^ mutant. **(C)** representative examples of cells after transfection; white lines indicate length of cellular extensions. **(D)** Model for mechanism of Sox2 action during NSC development.

Consistent with this, when the effect of ectopic Sox2 or the mutant Sox2 ^M203–209^ expression on GFAP and six genes in NSCs was assessed by qPCR, a range of levels of repression was exhibited by WT Sox2. This showed repression by WT Sox2 and a reduced or lack of repression by the Sox2^M203–209^ mutant (Figure 6A).

### Groucho-binding mutant of Sox2 fails to repress neural differentiation

Overexpression of Sox2 in NSCs has been shown to interfere with their ability to differentiate [18, 19]. We therefore compared the effect of overexpressing WT Sox2 to the Sox2^M203–209^ mutant. Unlike control NSCs transfected with EGFP alone, cells transfected with WT Sox2 did not extend fine, MAP2 positive processes after 5 days in differentiation medium, but instead, fewer cells extended broader, shorter processes (Figure 6B,C). However, no such inhibition of differentiation was seen in cells transfected with the Sox2^M203–209^ mutant.

## Discussion

Sox2 is a central component of the gene regulatory network that controls a range of stem cells, most notably ESCs and NSCs. The realization that, in addition to its role as a transcriptional activator, Sox2 might also repress genes is relatively recent and little has been done to investigate this activity.

Here, we have shown that the number of genes repressed by Sox2 in NSCs is almost identical to the number activated and we have identified one mechanism (recruitment of groucho family corepressors) by which it can achieve this repression. We have consequently generated a version of Sox2 that acts as a transcriptional activator but now lacks that mechanism for repressor activity. This allows us to begin to dissect the full complexity of Sox2 activity in regulating cell behaviour.

### Sox2 mechanism of repression

We chose to investigate a potential interaction with Grgs since this interaction has already been shown to mediate repression by the HMG family protein, Tcf [14, 15]. We have shown that Sox2 does indeed, directly interact with both full length and short forms of the Grgs. We used a series of deletions constructs to map the putative Grg-interacting region of Sox2 to amino acids 203–209 (YDVSALQY), which shows similarity to the Eh1 Grg interacting domain, F/YxI/VxxI/L/V [20, 21]. Targeted mutation of this region localized the interaction to the sequence, DxxxLQY, which is well conserved in the SoxB1 family.

However, alternative repressor mechanism(s) may also exist. This would provide multiple aspects to the mechanisms of action of Sox2 that could therefore be independently regulated to achieve a high level of complexity in its biological activities. It is also possible that some of the repressive effects of Sox2 are indirect via transcriptional activation of a repressor. Our results were over a short time scale so we do not feel that there was likely to be sufficient time for this to occur but it remains formally possible. This is supported by our earlier observations that the repressive effects of the very similar protein, Sox3, were mimicked by an HMG-engrailed repressor fusion protein [22].

### Relative role of transcriptional repression in Sox2 functions

Since Sox2 has traditionally been regarded as a transcriptional activator, it is striking that our study revealed that the number of genes repressed by overexpression of Sox2 was a similar to the number of genes that were activated. This implies that repression plays as big a part in its biological functions as does activation. Since the numbers of genes affected by Sox2 in our study closely resembles the numbers of genes affected when Sox2 was knocked down in ES cells [6, 7] it seems probable that the effects in our study represent true targets of Sox2.

It is of note that the targets of Sox2 activation are highly enriched for regulators of Pol II transcription whereas no such genes are repressed. This implies that a large part of the activator function of Sox2 (approximately 25% of the genes affected by Sox2 overexpression) is to regulate the biological activity of NSCs indirectly through the function of downstream transcription factors, whereas its repressor function affects the cells directly though regulating effector genes. Since Sox2 is expressed in dividing progenitor cells, the enrichment for cell cycle related genes in those repressed by Sox2 looks at first to be counterintuitive. However, this observation suggests that its normal role may be in part to control the rate of mitosis in those stem cells.

Previous studies have shown that the effects of SoxB1s in inhibiting the differentiation of NSCs was mimicked by a constitutive activator form of SoxB1 protein, while an HMG-EnR construct caused cells to begin to differentiate, suggesting that the effects of the SoxB1s were entirely through its activity as a transcriptional activator [18, 19]. However, overexpression of the HMG-EnR construct did not elicit complete differentiation as shown by the absence of neurofilament or beta-tubulin expression [18]. Indeed, close inspection of the published data shows that an HMG-VP16 construct inhibits expression of early neurogenic transcription factors, but does not appear to completely inhibit expression of beta-tubulin.

We therefore suggest a model in which the activator function of Sox2 promotes ‘stem cell-ness’ and so inhibits differentiation, but the repressor function of Sox2 is also required to inhibit differentiation, repressing those effector genes that would be activated soon after the cells were released from the NSC state (Figure 6C). Consistent with this model and the published data, the gene encoding neurofilament light chain was amongst those genes revealed to be repressed greater than 1.5-fold by Sox2 in our microarray analysis.

### Target specificity

Our observation that some genes are activated while others are repressed in the same transfected cell population, suggests that it is the gene target sequence that determines whether Sox2 exerts its activator or repressor activity. Activation or repression is not dictated solely by the availability of cofactors for these functions in a ‘cell context’ dependent manner, but is dictated by the target gene, which presumably determines which Sox2 cofactors are available at that regulatory region to cause Sox2 to act as either an activator or repressor.

## Conclusions

This study shows that transcriptional repression is a major part of the mechanism by which Sox2 acts in NSCs. In order to understand how Sox2 functions to regulate stem cell biology, we must therefore understand not only what is upstream and downstream of Sox2, but also which cell type-dependent cofactors are required for Sox2 to regulate each target and the gene sequence context that determines whether the target gene is activated or repressed.

## Methods

### Cell culture

The human ReNcell VM NSC line (Millipore Corp., Bedford, MA, U.S.A) was maintained as adherent cells in laminin-coated flasks (Sigma Cat. No. L-2020) with ReNcell NSC Maintenance Medium (Chemicon) supplemented with 20 ng/ml growth factors (bFGF and EGF, Invitrogen) in 50/50 mixture of Neurobasal and DMEM F12 (Gibco) medium supplemented with Penicillin/Streptomycin (Sigma), B27 and N2 (Gibco). P19CL16 mouse EC cells were maintained in Alpha Minimum Essential Medium (GIBCO) supplemented with 10% foetal bovine serum (FBS, Gibco) plus 0.5% Penicillin/Streptomycin. COS-7 cells were maintained in Dulbecco’s Modified Eagle’s medium with 10% FBS. Differentiation of NSCs was induced by removal of growth factors.

### Immunofluorescent staining

Cells were cultured on poly-D-lysine coated coverslips, fixed with 4% paraformaldehyde/PBS for 10 min and permeabilised with 0.2% Triton X100/PBS for 20 min. Blocking was carried out with 10% BSA in 0.1% Triton X100/PBS for 30 min. Primary antibodies (MYC antibody (9E10), Sox2 antibody (R&D, MAB2018), MAP-2 antibody (Abcam)) and the fluorescent-conjugated secondary antibodies were incubated at room temperature for 1 h. Staining was observed after mounting in mounting medium for fluorescence with DAPI (Vector). Error bars in Figures 1C based on counting 15 cells and 3A, and 4A counting 100 cells.

### Transfection

The human ReNcell VM NSC line (Millipore Corp., Bedford, MA, U.S.A) was transfected using the Mouse NSC Nucleofector® Kit (Lonza) following the manufacturer’s instructions. P19CL16 mouse EC cells were transfected using DharmaFECT transfection reagent (Dharmacon, Quiagen). COS-7 cells were transfected by square pulse electroporation; 210 volts for 50 mSec using a BTX Electro Square Porator ECM 830.

### Immunoprecipitation and immunoblotting

Two days after transfection, cells were treated with the cross-linker, 1 mM dithio-bis(succinimidyl propionat) (DSP; Sigma) for 30 min. The cells were lysed, cleared by centrifugation and incubated with MYC antibody-conjugated beads (Sigma). Eluted proteins were analysed by SDS-PAGE and western blotting using anti-MYC and anti-Sox2 antibodies (See supplementary material for additional details).

*In vitro* pull down assays were performed using GST-fusions of Grg1 and Grg5 (cloned into the pGEX4T1 vector). These proteins were induced in BL21 *E.coli* cells by the addition of 0.1 mM isopropyl-D-thiogalactopyranoside. After 16 h growth at 18°C bacteria were harvested and lysed. Solubility of GST-Grg proteins was increased by an additional 1 h incubation (4°C) in lysis buffer with 1% Nonidet P-40 and 0.03% SDS. GST-fusion proteins were subsequently incubated with S^35^-labelled Sox2 proteins produced *in vitro* using a TNT T7 kit (Promega), with (Amersham Biosciences). GST-fusion protein were pulled-down using Glutathione-sepharose 4B beads (G.E Healthcare) and the presence of co-precipitated Sox proteins assessed by exposure of a PAGE gel on a phosphorimager.

### Luciferase assay

The luciferase assay was carried out 24 h after transfection with the dual-luciferase-reporter assay system (Promega) following manufacturer’s instructions. Error bars represent standard deviation based on three independent transfection experiments.

### Expression profiling microarray

hNSCs were transfected with EGFP with or without pcDNA-Sox2 or pcDNA- Sox2^M203–209^. After 14 h culture, GFP-positive cells were isolated by FACS. Total RNA was extracted using TRI reagent (Sigma) and further purified using an RNeasy kit (QIAGEN). The RNA samples were analysed using GeneChip® Human Genome U133 Arrays (Affymetrix) and data were first preprocessed using the statistical software, R with packages provide by http://www.bioconductor.org. Data was preprocessed using the RMA method [23] and filtered such that probes which gave expression outputs below control background probes (recorded in the GeneChip) were excluded. Fold differences in expression were calculated, and annotation packages were used to assign gene information to each probe set. The data was exported as a .txt file in order to be read and analysed in Excel. The Excel tool PivotTable was used to assign average expression intensity values to each gene.

### Site directed mutagenesis

Point mutants were generated using the QuikChange kit (Stratagene) according to manufacturer‘s instructions.

### Quantitative RT-PCR

The extraction of total RNA from transfected hNSC using TRI reagent (Sigma) was carried out according to the manufacturer’s instructions. Total RNA samples were cleaned up using the RNeasy Mini Kit (Qiagen). Purified total RNA was used for the synthesis of cDNA using SuperScript III Reverse Transcriptase (Invitrogen). The primers used in the quantitative RT-PCR are listed below. Quantitative RT-PCR was carried out by Rotor-Gene 6000 (Corbett/Qiagen) with Brilliant SYBR Green QPCR Master Mix (Agilent). The PCR programme was set at 95°C for 15 seconds, 60°C for 20 seconds and 72°C for 20 seconds. All the quantitative RT-PCR data were analysed by Rotor-Gene 6000 real-time rotary analyzer version 1.7. The relative expression level compared to cyclophilin B was calculated according to Pfaffl [24]. Error bars in Figure 6A– standard deviation based on three replicated qPCR reactions.

Details of plasmids and primers available in supplementary material.

## Electronic supplementary material

Additional file 1: Table S1: Affymetrix expression data from GFP and Sox2 transfected human NSCs. Data from GeneChip® Human Genome U133 Arrays. Columns G and H are anti-logged values of columns E and F allowing un-logged ratios of expression to be clear, as shown in columns I and J. (XLSX 3 MB)

Additional file 2: Table S2: List of GO terms of genes that increased in expression when Sox2 was over-expressed in NSCs. N is the total number of genes; B is the total number of genes associated with a specific GO term; n is the flexible cutoff, i.e. the automatically determined number of genes in the ‘target set’ (ie. affected by Sox2 overexpression) and b is the number of genes in the ‘target set’ that are associated with a specific GO term. Enrichment is defined as (b/n)/(B/N). FDR q-value - False Discovery Rate analogue of the p-value. (TXT 775 KB)

Additional file 3: Table S3: List of GO terms of genes that decreased in expression when Sox2 was over-expressed in NSCs. N is the total number of genes; B is the total number of genes associated with a specific GO term; n is the flexible cutoff, i.e. the automatically determined number of genes in the ‘target set’ (ie. affected by Sox2 overexpression) and b is the number of genes in the ‘target set’ that are associated with a specific GO term. Enrichment is defined as (b/n)/(B/N). FDR q-value - False Discovery Rate analogue of the p-value. (TXT 346 KB)

Additional file 4: Figure S1: Nuclear localization of various Sox2 deletion mutants (B) and the Sox2 ^M203–209^ mutant (C) as compared to WT Sox2 (A). Sox2 proteins were overexpressed in COS-7 cells and visualized after 20 h using anti-sox2 antibody (green). Nuclei were counter stained with DAPI (blue). All proteins localized exclusively to the nuclei. Scale bar approximately 10 μm. (TIFF 138 KB)

## Abbreviations

CNS: Central nervous system
DAPI: 4’,6-diamidino-2-phenylindole
EGFP: Enhanced green fluorescent protein
enR: engrailed Repressor domain
ES cell: Embryonic stem cell
FACS: Fluorescent activated cell sorting
GO: Gene ontology
HMG: High mobility group
iPS cell: induced Pluripotent cell
NSC: Neural stem cell
PCR: Polymerase chain reaction
RT-PCR: Reverse transcription PCR
VP16: Activator domain from VP16 virus
WT: Wild type.

## References

[1] Yamanaka S, Blau HM (2010). Nuclear reprogramming to a pluripotent state by three approaches. Nature.

[2] Hanna JH, Saha K, Jaenisch R (2010). Pluripotency and cellular reprogramming: facts, hypotheses, unresolved issues. Cell.

[3] Graham V, Khudyakov J, Ellis P, Pevny L (2003). SOX2 Functions to Maintain Neural Progenitor. Identity.

[4] Rex M, Orme A, Uwanogho D, Tointon K, Wigmore PM, Sharpe PT, Scotting PJ (1997). Dynamic expression of chicken Sox2 and Sox3 genes in ectoderm induced to form neural tissue. Dev Dyn.

[5] Pevny LH, Nicolis SK (2009). Sox2 roles in neural stem cells. Int J Biochem Cell Biol.

[6] Masui S, Nakatake Y, Toyooka Y, Shimosato D, Yagi R, Takahashi K, Okochi H, Okuda A, Matoba R, Sharov A, Ko MSH, Niwa H (2007). Pluripotency governed by Sox2 via regulation of Oct3/4 expression in mouse embryonic stem cells. Nat Cell Biol.

[7] Greber B, Lehrach H, Adjaye J (2007). Silencing of core transcription factors in human EC cells highlights the importance of autocrine FGF signaling for self-renewal. BMC Dev Biol.

[8] Boyer LA, Lee TI, Cole MF, Johnstone SE, Levine SS, Zucker JP, Guenther MG, Kumar RM, Murray HL, Jenner RG, Gifford DK, Melton DA, Jaenisch R, Young RA (2005). Core transcriptional regulatory circuitry in human embryonic stem cells. Cell.

[9] Chen X, Xu H, Yuan P, Fang F, Huss M, Vega VB, Wong E, Orlov YL, Zhang W, Jiang J, Loh Y-H, Yeo HC, Yeo ZX, Narang V, Govindarajan KR, Leong B, Shahab A, Ruan Y, Bourque G, Sung W-K, Clarke ND, Wei C-L, Ng HH (2008). Integration of external signaling pathways with the core transcriptional network in embryonic stem cells. Cell.

[10] Kim J, Chu J, Shen X, Wang J, Orkin SH (2008). An extended transcriptional network for pluripotency of embryonic stem cells. Cell.

[11] Sharov AA, Masui S, Sharova LV, Piao Y, Aiba K, Matoba R, Xin L, Niwa H, Ko MS (2008). Identification of Pou5f1, Sox2, and Nanog downstream target genes with statistical confidence by applying a novel algorithm to time course microarray and genome-wide chromatin immunoprecipitation data. BMC Genomics.

[12] Brantjes H, Barker N, van Es J, Clevers H (2002). TCF: Lady Justice casting the final verdict on the outcome of Wnt signalling. Biol Chem.

[13] Eden E, Navon R, Steinfeld I, Lipson D, Yakhini Z (2009). GOrilla: a tool for discovery and visualization of enriched GO terms in ranked gene lists. BMC Bioinformatics.

[14] Brantjes H, Roose J, van De Wetering M, Clevers H (2001). All Tcf HMG box transcription factors interact with Groucho-related co-repressors. Nucleic Acids Res.

[15] Cavallo RA, Cox RT, Moline MM, Roose J, Polevoy GA, Clevers H, Peifer M, Bejsovec A (1998). Drosophila Tcf and Groucho interact to repress Wingless signalling activity. Nature.

[16] Shi W, Wang H, Pan G, Geng Y, Guo Y, Pei D (2006). Regulation of the pluripotency marker Rex-1 by Nanog and Sox2. J Biol Chem.

[17] Cavallaro M, Mariani J, Lancini C, Latorre E, Caccia R, Gullo F, Valotta M, DeBiasi S, Spinardi L, Ronchi A, Wanke E, Brunelli S, Favaro R, Ottolenghi S, Nicolis SK (2008). Impaired generation of mature neurons by neural stem cells from hypomorphic Sox2 mutants. Development.

[18] Bylund M, Andersson E, Novitch BG, Muhr J (2003). Vertebrate neurogenesis is counteracted by Sox1-3 activity. Nat Neurosci.

[19] Graham V, Khudyakov J, Ellis P, Pevny L (2003). SOX2 functions to maintain neural progenitor identity. Neuron.

[20] Goldstein RE, Cook O, Dinur T, Pisante A, Karandikar UC, Bidwai A, Paroush Z (2005). An eh1-like motif in odd-skipped mediates recruitment of Groucho and repression in vivo. Mol Cell Biol.

[21] Yaklichkin S, Vekker A, Stayrook S, Lewis M, Kessler DS (2007). Prevalence of the EH1 Groucho interaction motif in the metazoan Fox family of transcriptional regulators. BMC Genomics.

[22] Shih Y, Kuo C, Hirst C, Dee C, Liu Y, Laghari Z, Scotting PJ (2010). SoxB1 transcription factors restrict organizer gene expression by repressing multiple events downstream of Wnt signalling. Development.

[23] Irizarry RA, Hobbs B, Collin F, Beazer-Barclay YD, Antonellis KJ, Scherf U, Speed TP (2003). Exploration, normalization, and summaries of high density oligonucleotide array probe level data. Biostatistics.

[24] Pfaffl MW (2001). A new mathematical model for relative quantification in real-time RT-PCR. Nucleic Acids Res.

