# Supplementary figures and images for: Sox2 acts as a transcriptional repressor in neural stem cells
Source: BMC Neurosci. 2014 Aug 8;15:95. doi: 10.1186/1471-2202-15-95 (PMC4148960; doi:10.1186/1471-2202-15-95)

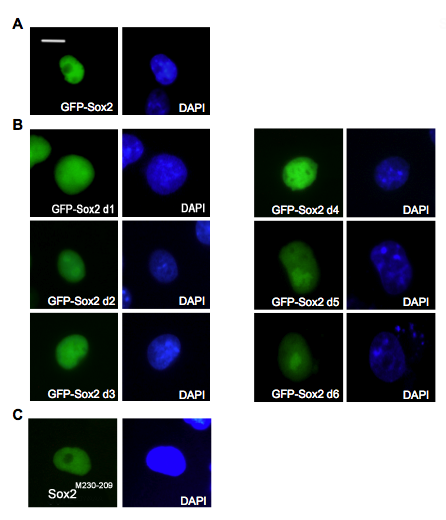

Supplement: Supplementary file 4 — Additional file 4: Figure S1: Nuclear localization of various Sox2 deletion mutants (B) and the Sox2 M203–209 mutant (C) as compared to WT Sox2 (A). Sox2 proteins were overexpressed in COS-7 cells and visualized after 20 h using anti-sox2 antibody (green). Nuclei were counter stained with DAPI (blue). All proteins localized exclusively to the nuclei. Scale bar approximately 10 μm. (TIFF 138 KB) [file 12868_2014_3786_MOESM4_ESM.tiff]
